# Recognition Gaps and COVID Inequality: The Case of Immigrants in Sweden

**DOI:** 10.1177/17499755231170700

**Published:** 2023-05-19

**Authors:** Andrea Voyer, Vanessa Barker

**Affiliations:** Stockholm University, Sweden; Stockholm University, Sweden

**Keywords:** COVID-19, cultural processes of inequality, immigrants, racialization, recognition gaps, Sweden

## Abstract

In this article, we examine recognition gaps exposed by the coronavirus pandemic. We apply Lamont’s cultural processes of inequality framework to the critical case of COVID inequality during the first wave of the pandemic in Sweden – a period in which COVID-19 cases were concentrated among immigrants. We identify recognition gaps associated with five key cultural processes of inequality. Counter to the dominant narrative of Sweden as an open and equal society, our analysis uncovers cultural processes of inequality theorists have identified in other contexts: the racialization of immigrants; and the stigmatization and evaluation of immigrant spaces. We identify two additional cultural processes: resignification in which the State’s coronavirus response was directed toward ethnic Swedish people; and inversion, in which higher death rates among immigrants were relabeled as a natural and acceptable cause of COVID deaths. In addition to applying and extending the theory, we demonstrate the value of a focus on recognition for studies of health inequality. The recognition gaps we identify in this article are practical and solvable problems. In comparison with the challenges of managing large-scale economic redistribution or abolishing prejudice and stigmatization by addressing bias on a person-by-person basis, anticipating and counteracting the cultural processes of inequality is an actionable pathway to pursuing more just and equal societies.

## Introduction

The COVID-19 pandemic was not merely a natural disaster, it was also a social disaster. The pandemic exposed deep structures of inequality across and within societies. More unequal societies experienced more COVID-19 deaths (Elgar et al., 2020) and the pandemic’s ‘racist morbidities’ (Murji and Picker, 2021) soon became apparent. Within and across countries, existing inequalities of class, race, ethnicity, citizenship, and prestige were reflected in the likelihood of contracting and dying from the virus, access to vaccines and treatments, inclusion in programs for economic recovery, and the right to travel across national borders (Barker, 2020; Bentley, 2020; Casaglia, 2021; Disney et al., 2022; Greenaway et al., 2020; Rostila et al., 2020; Usher, 2022; Yu et al., 2021). In other words, being a ‘minority’ or an ‘outsider’ was a common COVID risk factor shared by people from different racial and ethnic groups and with different migration histories who resided in different nations with different levels of inequality in and access to medical and social support systems. Sweden was no exception. This makes COVID inequality a good opportunity to uncover local cultural processes of social recognition, which are understudied but significant drivers of inequality including health inequality (Clair et al., 2016; Lamont et al., 2014).

In this article, we use the case of COVID inequality to show how cultural processes produce and reproduce inequality in ways that are not fully appreciated in Sweden. We further aim to advance theory on social recognition and the cultural processes of inequality, in particular concerning foreign-born people and their children, who make up the immigrant second generation (Alba, 2005; Honneth, 1995; Jaworsky, 2016; Lamont, 2018). Immigrants in this common sociological use is not synonymous with formal legal immigration status (Alba, 2005). In Sweden there is also a folk concept and symbolic category of *immigrant* (Hübinette and Lundström, 2014; Khayati, 2017; Voyer and Lund, 2020), which is described later in this article. We use italics when referring to this symbolic category.

Analyzing Sweden’s COVID inequality, we identify five key cultural processes associated with recognition gaps: (1) Racialization of immigrants; (2) Stigmatization of immigrant spaces; (3) Evaluation in which biased official categories devalued immigrant spaces; (4) Resignification in which the State’s coronavirus response was directed toward ethnic Swedish people, mistaking the symbolic center for the whole of society; and (5) Inversion, in which higher death rates among immigrants were labeled as a natural and acceptable cause of COVID mortality.

This analysis of COVID inequality demonstrates the benefits of attention to cultural processes of inequality. Particularly in a context known for its high quality of life, well-being, and comprehensive welfare state, inequality in COVID risk requires sociological examination. Analyzing the Swedish response to the crisis of the pandemic, we show how recognition gaps are institutionalized in policy and practice. Moreover, the recognition gaps we identify in this article are practical and solvable problems. In comparison with the challenges of managing large-scale economic redistribution or abolishing prejudice and stigmatization by addressing individual prejudice and bias on a person-by-person basis, anticipating and counteracting the cultural processes of inequality is an actionable pathway to pursuing more just and equal societies (Clair et al., 2016).

## Theory and Literature: The Cultural Processes of Inequality

Sociologists often focus on the unequal distribution of material resources as causes or drivers of social inequality, including health inequalities. While not denying the significance of material inequality, cultural sociologists point to the importance of symbolic social processes of boundary-creation, stigmatization, and exclusion (Lamont et al., 2014). Theorizing around the cultural processes of inequality is centered on social meaning, including individual attitudes and understandings, but also shared meanings such as social norms and cultural scripts linking individual cultural elements like taste, choice, intention, and aspiration with macro-level structures of inequality such as neighborhood effects and the intergenerational transmission of advantage and disadvantage (Lamont et al., 2014: 579–82).

### Social Recognition

Lamont and many collaborators have developed theory and a research program on cultural processes of social inequality. The work includes such elements as symbolic social boundaries (Lamont and Molnár, 2002), processes of legitimation and evaluation (Lamont, 2012), and stigmatization (Lamont et al., 2016). The theory is unified around recognition (Lamont, 2018: 423) – that identifying and countering ‘recognition gaps’ opens up new possibilities for the pursuit of more equal societies. If and when people are seen or not seen in society matters to life chances. To be recognized and acknowledged as a member of society is fundamental to human interaction and social organization (Honneth, 1995).

Social recognition refers to the processes by which some ways of life are embraced as the socially and morally correct norms and practices of society, while recognition gaps refer to processes by which other ways of life and the people who practice them are ignored or condemned. Persistent inequalities are not natural. An individual’s social status does not arise intrinsically from cultural advantages or disadvantages, it arises *relationally* through processes of social recognition. Recognition, therefore, forms the underlying structure of social relations: How do we know we are a society? How do we know as individuals we are part of a society? Who is not part of society? These are basic but profound questions that provide sociology its disciplinary backbone and are central to explaining persistent inequalities. Who is in and who is out? Who decides? On what terms? To be ignored, outside, and invisible is a specific kind of social exclusion and stigmatization. Recognition is the counterpoint to cultural processes of inequality, and the establishment of inclusive social membership is the goal of recognition efforts (Lamont, 2018: 422, 419).

### Recognition Gaps

*Recognition gaps* occur when there is a failure to recognize certain social groups as full members of society (Lamont, 2018). Cultural processes unfold within two dimensions: identification processes by which individuals and groups are categorized and situated in within broader collective groupings, and rationalization processes in which biased, but purportedly neutral, routines and practices are created and implemented (Lamont et al., 2014). The processes unfolding in these two dimensions include *racialization* (in which social and phenotypical markers become significant indicators of essential difference), *stigmatization* (the process of attaching negative significance to characteristics), *standardization* (the construction and application of presumably neutral and uniform norms and rules), and *evaluation* (presumably neutral categorizations and assessments of value or worth) (Lamont et al., 2014). This set of cultural processes accounts for a wide range of cases and contexts of inequality, but it is not exhaustive (Lamont et al., 2014: 597) or necessarily sequential. In our case, these processes are iterative and tend to compound one another, which led to the discovery of two additional cultural processes: *resignification* and *inversion*, described later.

The cultural processes of inequality framework draws a clear contrast with culture-of-poverty approaches based on assumptions of cultural deficiency. These approaches highlight self-perpetuating cycles of inequality driven by neighborhood or group practices, family structure, and the socialization of youth to ‘problematic’ values and norms and rendering some groups incapable of social and economic mobility (Wilson, 2012 [1987]). Unlike the cultural processes framework, such explanations are not designed for observing ongoing structural conditions contributing to cycles of poverty (Wilson, 2012 [1987]) or continued discrimination and the reproduction of advantage through recognition gaps (Greenbaum, 2015; Lamont et al., 2014).

### Cultural Processes of Health Inequality

The cultural processes of inequality framework have already been applied to the topic of health inequality. Clair et al. (2016) show that stigma reduction efforts reducing negative views of social groups at the center of the HIV and obesity epidemics can have important impacts on responses to public health problems. Meanwhile, Asad and Clair (2018) argue that stigmatized legal statuses (i.e. being associated with a criminal record and undocumented immigrant status) have negative impacts on health for both individuals that hold and those that are expected to hold those statuses. This article contributes to this area of research with an analysis of recognition gaps exposed in the case of COVID inequality during the onset of the pandemic in Sweden.

## Background: COVID Inequality in the Swedish Welfare State

Sweden’s COVID inequality is a *critical case* (Flyvbjerg, 2011) for observing cultural processes of inequality because the comprehensive Swedish welfare state limits material inequality in comparison with many contexts (e.g. less inequality in access to healthcare than places without national healthcare). Relative to contexts with less inequality-reducing infrastructure, COVID inequality in Sweden makes cultural processes of inequality more visible.

### The ‘Swedish Miracle’?

Known for its welfare state and emphasis on equality and human rights (Schaffer, 2020), Sweden ranks among the highest OECD countries in terms of quality of life and health of its democracy (OECD, 2020). Sweden provides national health insurance for all legal residents. Sick leave and other vital social supports are widely available. These social supports are intended to reduce inequality. Sweden’s social safety net is something that both foreigners and Swedish citizens recognize as a special characteristic of the country (Simons, 2020; Smith, 2006).

Sweden is also ethnically and racially diverse. Beginning in the 1990s, large-scale migration of refugees and asylees has shifted Sweden’s population demographics. In 2020, over 20% of the nearly 10.4 million people in Sweden were immigrants with ‘foreign background,’ being either born abroad themselves or with one or more parents born abroad (Statistics Sweden, 2020). The national origins of Sweden’s immigrant population are diverse. In 2021, the top 10 sending countries for foreign-born people in Sweden were Syria, Finland, Poland, Iran, Somalia, Afghanistan, the countries of the former Yugoslavia, Bosnia and Herzegovina, and Turkey. The top 10 foreign backgrounds of the Swedish-born immigrant second generation where both parents have the same national origin were Finland, Iraq, Syria, Somalia, Yugoslavia, Turkey, Iran, Bosnia and Herzegovina, Poland, and Lebanon (Statistics Sweden, 2022).

The Swedish welfare state is not as strong as it once was. Beginning in the 1990s, a program of neo-liberal reforms was introduced and the social safety net was redesigned (OECD, 2015). As a result, Sweden has growing economic inequality, especially for long-term unemployed persons and newly arrived immigrants (Therborn, 2020). While some attribute increased inequality and its immigration link to neoliberal reforms and welfare state restructuring (Schierup and Ålund, 2011), others emphasize on the mechanisms of class reproduction (Hällsten and Pfeffer, 2017), labor market dynamics, including discriminatory hiring and recruitment practices favoring native Swedes (Bursell, 2014), the long-term effects of housing segregation (Backvall, 2019), disparities in educational attainment, and exclusion from the democratic process (de los Reyes et al., 2014; Hörnqvist, 2016). These factors are found to contribute to the rise and persistence of inequality in Sweden and surely account for some of the health differentials we see with COVID-19 (Drefahl et al., 2020). However, they all point to material inequality. In this article, we highlight the understudied cultural processes linked to recognition gaps.

### COVID in Sweden

As Sweden’s first cases of COVID-19 were reported in January 2020, government officials and public authorities expressed optimism that the virus would be contained (see Rodan, 2020). The Swedish Public Health Agency (FHM), which was tasked with developing the nation’s coronavirus response, published routine reports, public guidelines, and forecasts. They conducted regular press conferences to share this information, which documents the slow rise in COVID cases connected to foreign travel and managed through contact tracing. COVID-19 was a leading news item and a prevalent topic of public conversation.

The optimism faded quickly. The surge of COVID cases in northern Italy coincided with Sweden’s February school holiday – a time when many people travel to Italy (FHM, 2020a). The number of known cases linked to international travel rose quickly, and in early March, the first cases with no clear link to international travel and the first death were reported (see Claesson, 2020; Erlandsson, 2020). In mid-March, FHM announced a new phase in the pandemic. Instead of stamping out coronavirus through contact tracing, the new goal was slowing the spread of the virus and protecting the most vulnerable populations – a ‘risk group’ comprising seniors and people with underlying health conditions (FHM, 2020b). Nursing homes and hospice centers were placed on lockdown. All who could were asked to work from home. High schools and universities moved to remote instruction.

Sweden became known for its lax approach to the coronavirus, emphasizing social distancing, bans on large gatherings, and personal responsibility instead of mandating the widespread use of face masks, mass testing, and lockdowns (Simons, 2020; Yan et al., 2020). Sweden’s excess mortality rate rose quickly, outpacing neighboring Norway, Denmark, Iceland, and Finland (Yarmol-Matusiak et al., 2021). Even though Sweden was an outlier in terms of the relaxed policies and COVID statistics, there was little dissent regarding FHM’s policies. Unlike many other nations, the pandemic was not politicized in Sweden (Sparf et al., 2022). To account for Sweden’s outlier approach and its broad public support, most expert commentaries focused on high trust in government, civic pride, a dominant (if not absolute) consensus culture, and the public health authority’s unusual power to determine policy uninfluenced by politics (e.g. Trägårdh and Özkirimli, 2020).

### COVID Inequality

Figures on COVID-19 infection and mortality during the first wave of the pandemic in Sweden showed serious discrepancies: foreign-born people were over 200 times more likely to contract the virus than natives (FHM, 2020d). Between 31 January and 4 May 2020, Swedish residents born in the Middle East and Africa were about three times more likely to die of COVID-19, while even those born in other Nordic countries were 50% more likely to die than Swedish-born individuals (Rostila et al., 2020). Those at the highest risk for COVID mortality were people born in Somalia (risk 9 times higher than Swedish-born individuals), Lebanon (6 times higher) Syria (5 times higher), Turkey (3 times higher), Iran (3 times higher) and Iraq (2 times higher) (Rostila et al., 2020). These disparities persisted as the pandemic continued, although in muted form (Andersson et al., 2021).

## Method

COVID inequality between immigrants and natives in Sweden is a critical case (Flyvbjerg, 2011) and following case study methods (Yin, 2009), we rely on different kinds of data, including information on COVID-19 and the social context available from government documents, official statistics, government statements, national and local media accounts, and public health warnings and recommendations, as well as our observations and interpretations as people experiencing the pandemic in Sweden. We were in the Stockholm region during the pandemic. As sociologists who live and work in Sweden, but relocated from the USA, we drew on our backgrounds as analytical leverage to observe cultural processes that may be less apparent to cultural insiders (for example, as described later, the reliance on Swedish cultural competency in health recommendations), but we also used our knowledge of Sweden to contextualize the pandemic within Swedish history and society.

We began data collection in March 2020, when COVID inequality became visible. We incorporated primary material from as early as January 2020. We concluded the main data collection in May 2020, corresponding with the decline in cases at the end of the first wave of the pandemic in Sweden (Kawalerowicz et al., 2022). During this period, we observed the regular press conferences from the public health authority, FHM, as well as addresses from leaders of the Swedish government and the monarchy. FHM posted statistics, reports, strategy, communications, and other relevant material on its website daily. We also read the national newspapers’ coverage of the pandemic, including both news reporting and editorial pieces. To contextualize the material and support the analysis, we drew on relevant information from earlier and later periods – including official statistics, historical accounts, secondary sources, and data on immigration. We also compared what was happening inside Sweden to what was happening outside of Sweden – for example, the politicization of the pandemic response in the USA (Villegas, 2020) and the role of rigid lockdowns in most other European nations (Yan et al., 2020).

The research is limited to the first wave of the pandemic. As Klinenberg (2002) has shown, public health crises often expose deep social fault lines that are not always observable. The initial government response to the pandemic exposed cultural rifts in Sweden that may not have been visible otherwise. Subsequent research could observe additional recognition gaps emerging as the situation developed. Some of the recognition gaps we describe were addressed quickly while others persisted as the pandemic continued.

In analyzing the material, we use abductive logic (Swedberg, 2012), meaning our analysis is informed by our theoretical emphasis on recognition gaps and by the data itself. COVID inequality between native and non-native Swedes pointed us to the significance of the distinction between ‘immigrants’ and ‘Swedes’ as it was related to cultural processes of inequality. Our approach to the analysis was holistic and emergent (Lareau, 2021). We categorized the material thematically and identified different recognition gaps, as presented in the analysis. We worked with a large body of material, triangulating and corroborating information across multiple sources. For example, a comment the Prime Minister made in a prime-time television address to the nation might also be covered in the newspapers the next day and discussed in FHM’s morning press conference. Examining all of these things made it possible to establish the character of the Prime Minister’s remarks, and also see how they were interpreted. Here we present key and illustrative material.

## Analysis

The analysis exposes and makes visible five cultural processes of inequality resulting in recognition gaps: racialization; stigmatization; evaluation; resignification; and inversion. We describe these processes in turn.

### Racialization of Immigrants

Racialization refers to process by which social and phenotypical markers derive significance as indicators of essential difference (Lamont et al., 2014). In contemporary Sweden, *immigrant* is a racialized category, and we italicize this term when we intend to denote *immigrant* in this sense. Stigmatization and racism in Sweden tend to operate through a distinction between native *Swedish* (italicized here to represent a symbolic ethno-racial category) people and *immigrants* (Hübinette and Lundström, 2014; Khayati, 2017; Voyer and Lund, 2020). Non-native and/or non-white people who speak with foreign accents or otherwise fail to convey a sense of *Swedishness* through their attire, neighborhood, and social networks are typically referred to as ‘immigrants’(*invandrare*) or persons with ‘immigrant background’ (*utländsk bakgrund*) (Lund and Voyer, 2019). This distinction between *immigrant* and *Swedish* (*svensk*) is a symbolic ethno-racial distinction that is not equivalent, but is related to, formal legal categories.

Racialized group differences are seen as essential or fundamental (Lamont et al., 2014). *Immigrants* in contemporary Sweden are generally seen as incapable of being *Swedish*, and their assumed foreignness is documented in official population registries and cultural frameworks of understanding and practice (Barker, 2017). As Statistics Sweden (2020) reports, ‘most immigrants are Swedish-born’ with parents or grandparents who immigrated to Sweden (Lund and Voyer, 2019). Swedish-born children of *immigrants* are still called ‘immigrants’ or referred to as ‘new Swedes’ (*nysvenskar*). Meanwhile, people who are literal immigrants from majority white and western nations such as Finland, Norway, the UK, and the USA are generally not considered *immigrants* at all, even if they are included in official immigration statistics (Voyer and Lund, 2020).

#### Colorblind Ideology

In Sweden, the category *immigrant* includes different races and ethnicities, nationalities, religions, and other significant social and cultural groupings. While these groupings also have social meaning that could be explored, we chose to focus specifically on *immigrants* (versus *Swedish*) because the racialization of *immigrant* takes place in the context of a strong colorblind ideological commitment combined with a history of racial hierarchy associated with Sweden’s few official minority groups: Sami, Roma, Jews, Swedish Finns, and Tornedalers. These groups, recognized as having long historical ties to Sweden, receive government support to preserve their language and culture (Swedish Institute, 2022).

Sweden’s national minorities have not always been recognized and supported. Historically established hierarchies of racial differences included scripts of Nordic white superiority and beauty, civility, intelligence, and morality in comparison with Finns and the indigenous Sami (Kjellman, 2013). These views laid the pseudo-scientific groundwork for a national eugenics institute (SIRB, the Swedish State Institute for Racial Biology), a program of forced sterilization (Broberg and Tydén, 1996), and the forced removal and assimilation of some Sami children. Racial biology, discredited after World War II, did not completely disappear. The SIRB, renamed the Institute of Medical Genetics, continued racial surveys of the population through the post-war period (Ericsson, 2021).

In contemporary times, a desire to recover from this shameful past resulted in an authoritarian colorblind ideology (Hubinette and Tigervall, 2009). Racial, ethnic, and cultural differences are perceived as a threat, and not something easily incorporated into Swedish national identity (Carlsson et al., 2012). The State is prohibited from collecting data on race or ethnicity (Wikström and Hubinette, 2021). There is no national census in Sweden in which individuals self-report their ethnic or racial categorization. Instead, statistics are collected on national origin. In public life and research, focusing on specific racial and ethnic groups or racial and ethnic identity is considered to be racist (Voyer and Lund, 2020). Nevertheless, as in other countries, immigrants in Sweden tend to identify with their nationality or ethnicity, connect with others with the same identity, and form organizations based on these shared identities (e.g. Bayram et al., 2009; Carlsson et al., 2012). However, unlike other countries where the State may embrace immigrant organizations and apply the same logics of language and cultural preservation to both immigrant and indigenous groups (Bloemraad, 2006), in Sweden immigrant-group-specific identifications and mobilizations are considered problematic signs of ‘failed integration’ and group-specific organizations are often met with skepticism, possibly stymying immigrant incorporation (Carlsson et al., 2012).

This colorblind, anti-group approach creates a gap between imposed ideology and social reality. Researchers struggle to study racism within the constraints of official categories and researchers have long called for a more direct discussion of race and ethnicity in Sweden (Hubinette and Mählck, 2015; McEachrane, 2014; Schclarek and Mulinari, 2020). The continued use of *immigrant* as an overarching category, while central to ethnic diversity in Sweden, often obscures more than it reveals about how difference is valued, communicated, and incorporated in Swedish society. The racialization of *immigrants* in Sweden is one of the recognition gaps exposed in the case of COVID inequality. Stigmatization is another recognition gap.

### Stigmatization and Evaluation of Immigrant Spaces

*Stigmatization* is the process of attaching negative significance to characteristics associated with a particular category (Lamont et al., 2014). Although we observe stigmatization in relationship to a variety of practices, such as ways of speaking Swedish, ways of dressing, and choice of music, which are socially devalued because of their association with *immigrants*, in terms of COVID inequality the stigmatization of *immigrant* neighborhoods is the most glaring recognition gap.

Stockholm is one of the most segregated urban areas in Sweden, a country with one of the highest rates of residential segregation among OECD countries (Koopmans, 2010). White middle-class *Swedes* and western ex-pats tend to live in separate neighborhoods, more often inside the city centers or leafy suburbs, whereas *immigrants* tend to live in housing projects that ring the city centers. These concrete suburbs were built in the 1960s as part of the Million Program, an ambitious public housing project for the working class. Over the decades, the aging infrastructure of these neighborhoods and the rising popularity of home ownership and stand-alone houses led to lower rents and higher vacancy rates (Nesslein, 1982). Newly arrived immigrants are more likely to find housing in these areas, and those who struggle economically are more likely to remain (Andersson and Bråmå, 2004).

Minority spaces are often seen as undesirable and problematic (Voyer, 2019). Indeed, residential segregation in Sweden, as in most places, relies more upon *Swede*s’ avoidance of *immigrant* neighborhoods than upon the self-segregation of *immigrants* (Muller et al., 2018). Neighborhood stigmatization is felt by residents, who voice concerns about being treated differently, being excluded, disrespected by the police, and demeaned by public authorities (Schierup et al., 2014). Research documents a growing sense of frustration among residents of Sweden’s segregated neighborhoods (del los Reyes et al., 2014).

#### Evaluating Immigrant Neighborhoods

Recognition gaps also arise as stigmatized *immigrant* spaces undergo evaluation. The cultural process of evaluation is the assessment of value or worth based on presumably neutral categories (Lamont et al., 2014). Sweden’s official category of ‘vulnerable area’ (*utsatta områden*) is the basis for evaluation as a cultural process of inequality. The designation of vulnerable area is assigned by law enforcement and based on a variety of characteristics, including having lower socioeconomic status, lower education attainment, higher unemployment, a larger at-risk youth population, and higher crime rates (see Polisen, 2018). But this category is not neutral. Being outside of the *Swedish* norm is an explicit element of the ‘vulnerable’ designation, which also includes such characteristics as possessing parallel social structures such as the national and cultural group-specific organizations discussed earlier, having lower levels of Swedish language proficiency, and elevated risk of extremist religious views and the possibility for residents to sympathize with or participate in conflicts abroad – the official discussion of these last two concerns explicitly references practitioners of Islam, and the foreign conflicts associated with the Islamic State (IS) and al-Shabaab (see Polisen, 2018).

The stigmatization of *immigrant* neighborhoods and the formalization of that stigma through the assignment of a formal, supposedly neutral label only further the boundaries between *immigrants* and *Swedes*. There are social problems, including gun violence, the drug trade, and other criminal activities in many low-income Swedish suburbs, and these issues could benefit from increased spending on social programs and law enforcement, which is provided to ‘vulnerable’ neighborhoods (Polisen, 2018). However, the categorization of ‘vulnerable’ through a biased process of evaluation reinforces stereotypes about *immigrants*’ perceived failures to integrate. As a result, neighborhoods placed on the list of vulnerable areas often protest the designation despite the increased services (e.g. By, 2019).

#### ‘Segregation Kills’

Racialization, stigmatization, and evaluation are cultural processes of inequality relevant to COVID inequality. As discussed by FHM and reported in the media, in March 2020, neighborhoods with more immigrants, suburbs where as many as two out of three residents were born abroad, were feeling the brunt of the virus (see Berg and Skoglund, 2020; Hurinsky and Carp, 2020; Randhawa, 2020). Likewise, immigrants living in segregated neighborhoods were overrepresented in risk and mortality statistics (see Franssen, 2020; Nordström et al., 2020).

Structural conditions surely contributed to COVID inequality. Higher residential density, more multi-generational households, and poorer public health were routinely cited as driving the disparity (e.g. Berg and Skoglund, 2020; Franssen, 2020; Randhawa, 2020). But recognition gaps were at play as well. In the words of Ahmed Abdirahman, director of the Global Village Foundation, ‘Segregation kills. Corona kills too, but faster’ (Global Village Foundation, 2020). While the first wave unfolded, COVID inequality revealed additional cultural processes contributing to recognition gaps between *immigrants* and *Swedes.*

### Resignification: Sweden is for Swedes

Resignification, a cultural process of inequality first identified in this research, refers to recognition gaps in which the focus of attention is directed away from stigmatization and its impacts and back toward the symbolic center that remains. In this way, the center is mistaken for the whole of society. In the Swedish case, the contrast between *Swedish* and *immigrant* is implicit in the stigmatization and evaluation of *immigrant* spaces. As *immigrants* are stigmatized, they are susceptible to being treated as outsiders and nonmembers of society. Through resignification, the symbolic space of people who are not*immigrants* and spaces that are not ‘vulnerable’ are recast as the people and places of Sweden while those who are excluded fade into the background. When it comes to COVID inequality, resignification is evident in pandemic planning and pandemic policy.

#### Pandemic Planning

Following the first coronavirus death – an immigrant residing in a ‘vulnerable’ neighborhood, media outlets began discussing the lack of information about coronavirus in languages other than Swedish (e.g. Berg and Skoglund, 2020; Sundkvist and Anderberg, 2020). These reports noted that people who do not speak Swedish, more than 10% of the national population, had difficulties finding official information about COVID-19. At this point, information was provided in Swedish and sometimes English, but not the many other languages spoken in Sweden. Given limited official information, people turned to local institutions like immigrant aid societies, religious organizations, and senior centers. However, according to the reports, there had been no official outreach to these voluntary community organizations (e.g. Sundkvist and Anderberg, 2020).

The lack of accessible messaging could be due to the short time frame for pandemic response instead of cultural processes of inequality unfolding through the resignification of *Swedish* people as the people of Sweden, but the evidence suggests otherwise. In the face of criticism, the public health authority acknowledged that, although they began preparing for COVID-19 and related scenarios well in advance, there was no plan for outreach to non-Swedish-speaking populations and the neighborhoods where these populations were concentrated (see Sundkvist, 2020). More than an oversight, this failure to send the message to minority populations put FHM out of compliance with UN and World Health Organization’s guidance for risk communication and community engagement around COVID-19 (RCCE, 2020). Two weeks after the reports of language-accessibility problems and 10 days after the first COVID-19 death, FHM corrected its error. More than 6000 notices in 24 languages describing the dangers of COVID-19 and how to limit the spread of the illness were posted throughout virus-stricken neighborhoods as part of a large information campaign (see Ahmed, 2020; Bergman and Jobe, 2020; FHM, 2020c). But by that point, the health impacts were clear. As widely reported by FHM and the media, most of the first cases of community transmission involved *immigrants*, and 6 of the first 15 people who died were Somali immigrants (e.g. Berg and Skoglund, 2020).

#### Pandemic Policy

Resignification of *Swedish* people as the people of Sweden also extended to the way COVID-19 mitigation policies were formulated. Instead of clear rules, health guidelines were delivered as *recommendations*. For example, it was recommended to work from home. One should ask oneself if it was necessary to take mass transit or decide for oneself if one should go to the gym or meet friends at a restaurant. It was the individual’s responsibility to make the right choice. When pushed for clarity on how to make such decisions, for example during press conferences, public officials argued that the flexibility of recommendations worked well in the Swedish context.

The resignification of *Swedish* people as the people of Sweden is evident in the assumed level of cultural consensus and understanding on the part of the people the health recommendations were for. In late March, Prime Minister Löfven addressed the nation regarding the coronavirus. Löfven explained that every Swedish resident should follow the health guidelines and rely upon *folkvett* – a term translated by an English-language news service as ‘common sense manners’ and ‘the moral sense that every person is expected to have without being taught, and a word every Swede will instinctively recognize as something seen as a very, very bad thing not to have’ (Löfgren, 2020). When asked to explain more precisely how his agency wanted people to behave, the head of FHM, Anders Tegnell, said, ‘What we are talking about here is the Swedish culture, how Swedes interpret recommendations from the authorities. I think most people see [a recommendation] as very clear advice on how to do this in the best possible manner’ (see Rothschild, 2020). In other words, deciphering the guidelines that made up the heart of coronavirus mitigation efforts did not just require the ability to speak Swedish, it required *Swedish* common sense.

Not everyone had access to the knowledge necessary to interpret Sweden’s COVID-19 guidelines. An unscientific survey of international residents, including elite ex-pats employed as academics, creatives, and IT workers in addition to racialized *immigrants*, found overwhelming uncertainty around what one should do to mitigate the spread of the virus. Lacking clear guidance, people reported following the policies of their home countries. In the case of people coming from other western nations, home-country coronavirus restrictions were more stringent (see Edwards, 2020).

Resignification is a cultural process of inequality revealed in pandemic planning and policy in Sweden. From the beginning, COVID policies were designed with *Swedish* people in mind. Racialized and stigmatized *immigrant* populations and neighborhoods were neglected, but so were the elite ex-pats and international visitors living alongside Swedish people but lacking the required linguistic and cultural competence. Resignification is a process that re-naturalizes and essentializes the Swedish population as a homogenous entity, rendering its actual population invisible, with serious ramifications for public health policy and practice.

### Inversion: Immigrants Causing COVID Inequality

The final recognition gap is the process of inversion, newly identified in this case. Through inversion, inequality becomes a reason or a cause that explains its own effects. We observed two instances in which sense-making around Sweden’s COVID inequality was turned upside down and *immigrants* were blamed for COVID inequality. First, COVID inequality was interpreted as evidence of *immigrants*’ cultural deficiencies. Second, *immigrants*’ greater risk was presented as a cause of the country’s higher death rates.

#### Immigrant Culture

As the disproportionate suffering of *immigrants* became apparent, speculation arose as to the reasons. Some argued that ‘cultural aspects’ played a role (e.g. Busch, 2020a; see Franssen, 2020; Randhawa, 2020). In more benign interpretations, cultural explanations highlighted cultural differences in a matter-of-fact and value-free way. For example, Jihan Mohamed, a doctor on the board of the Somali-Swedish Medical Association, noted that ‘In Somali culture, it is important to socialize, support and visit each other, especially if someone is ill’ (Randhawa, 2020). Others discussed the value placed on multigenerational households and caring for the elderly at home (see Berg and Skoglund, 2020).

Initial value-neutral considerations of cultural factors were quickly overshadowed by, on the one hand, pointed criticism of the public health vulnerability of Sweden’s immigrants, and, on the other hand, a cultural process of inversion in which culture of poverty arguments attributed the suffering of *immigrants* to their own cultural failings. Culture of poverty explanations of COVID inequality emerged in the form of online expressions of hate. Some of these expressions celebrated the deaths as a way to decrease the minority population (see Jobe, 2020). These cruel and xenophobic sentiments were soon sanitized and weaponized as cultural essentialism.

For example, Ebba Busch, the leader of the far-right Christian Democratic party (KD), penned an opinion piece for the mainstream newspaper, *Aftonbladet*. In it, she argued that more deaths occurred among *immigrants* because they were ‘vulnerable.’ Here she used the term *utsatta*, which means vulnerable but also isolated or set apart – the same term used to refer to ‘vulnerable’ neighborhoods. Busch acknowledged the underlying risk factors noted by others, such as overcrowded and intergenerational households, but she classified these factors as problematic ‘cultural causes’ (*kulturspecifika orsaker*) and connected them to other cultural characteristics she attributed to *immigrants*: distrust of authorities, illiteracy, and the idea that medical advice is transmitted word of mouth instead of coming from experts (Busch, 2020a).

The process of inversion is clearly evident in Busch’s account. As she described it, the only blame the Swedish government and society bore for *immigrants* suffering was the crime of open borders. Busch concluded that COVID inequality was caused by the fact that ‘20% of immigrants in the country were not admitted with due consideration of their *integrationspotentialer*,’ meaning their potential for integration based on cultural similarity with Swedishness (Busch, 2020a, 2020b). Busch suggests that immigrants created COVID inequality, rather than seeing it as a part of enduring social problems linked to underlying structures of social recognition. This inversion of COVID inequality could be dismissed as a far-right perspective, but how different was it from the official view of the situation?

#### COVID Inequality as an Explanation

Inversion was also evident in the way government officials explained and justified Sweden’s COVID deaths. In September 2020, well after the first wave of the pandemic had subsided, FHM’s Tegnell was asked why COVID-19 had killed so many people in Sweden in comparison with neighboring Finland. Tegnell explained that Finland had ‘better conditions’ to contain the virus. He highlighted the country’s relative lack of urban density, the relatively limited international travel of Finland’s population, and the fact that the country has ‘almost no immigrant groups’ (see Svahn and Hallgren, 2020). We observed Tegnell offer this explanation multiple times when questioned about Sweden’s higher death rates.

Tegnell misrecognized COVID inequality as an explanatory factor instead of a factor to be explained. This inversion process, when combined with the racialization and stigmatization keeping *immigrants* outside or on the periphery of Swedish society, made it possible to present COVID inequality as an acceptable cause of deaths instead of an unacceptable effect of processes of social inequality. Tegnell was eventually criticized for blaming Sweden’s high COVID-19 death toll on immigrants. When faced with criticism, Tegnell apologized for his poor word choice (see Zangana, 2020), but he did not abandon the claim that Sweden’s larger immigrant population contributed to the country’s lackluster COVID-19 statistics.

Ultimately, inversion obscured the State’s responsibility for protecting *immigrants*. This is evident in contrast to the sense of shared responsibility punctuating discussions of the deaths of elderly people in nursing homes and hospice care. Tegnell, Prime Minister Löfven, and others acknowledged the heavy toll the virus took on this population, the work being done to address the problem, and the fact that protecting the elderly had been a goal from the beginning (see Bengtsson, 2020; Kerpner and Fernstedt, 2020; Svahn and Hallgren, 2020). While this failure was eventually the subject of a scathing coronavirus commission report, no national investigation of COVID inequality as it related to immigrants was undertaken and the coronavirus commission did not pick up the issue of COVID inequality associated with immigrants as an area for improvement (see Corona Commission, 2020, Corona Commission, 2022).

## Discussion: Recognition Gaps and COVID Inequality

Recognition gaps arise when some people in a community, society, or country are not recognized as part of ‘the people’. When the State and the citizenry mobilize symbolic boundaries of belonging in response to a crisis, recognition gaps are particularly visible and salient. Our analysis of COVID inequality in Sweden finds recognition gaps associated with the racialization of *immigrants* and the stigmatization and formal devaluation of *immigrant* neighborhoods before the spread of COVID-19. COVID’s ‘racist morbidity’ (Murji and Picker, 2021) was then reflected in recognition gaps evident as the pandemic unfolded. Ethnic *Swedish* people were resignified as the focus of government efforts to protect the people of Sweden. These resignification processes resulted in failures to recognize and plan for the needs of people outside of the *Swedish* herd. There was initially a lack of messaging for those who could not speak Swedish. When warnings and protections finally did arrive, they required ‘Swedish’ cultural competence to be comprehended. Recognition gaps were further evident in inversion. Instead of recognizing COVID inequality as a social problem to be addressed, the suffering of *immigrants* prompted a hostile public reaction. Conservative voices interpreted the COVID inequality as evidence of *immigrants*’ cultural deficiencies and lack of potential for integration into Swedish society, while public authorities argued that immigrant deaths could explain the country’s death rates. Given the relatively large immigrant population, the country’s higher death rate could be excused. To date, there has been no official investigation into the recognition gaps revealed by COVID inequality in Sweden, but there has been public acknowledgment of failures to protect other populations (e.g. Corona Commission, 2020).

## Conclusion

It is essential to consider recognition gaps when examining how societies respond to global and local crises, including disease pandemics (Klinenberg et al., 2020). The cultural processes of inequality framework makes it possible to identify recognition gaps arising when certain social groups and individuals fall outside of the ‘symbolic boundaries of belonging’ (Jaworsky, 2016) in a society, contributing to disparities in social worth. Attention to these cultural processes can help us better understand the breadth and depth of social inequality in the case of crises like the pandemic, and the routine functioning of unequal societies as well.

The COVID-19 pandemic exposed deep structures of inequality across and within societies. Examining the onset of the pandemic in Sweden, we observed five distinct cultural processes. These cultural processes exacerbate existing inequalities as recognition gaps reproduce hierarchies of human worth that facilitate other recognition gaps. Three of these processes were previously identified in the literature on cultural processes of inequality: the *racialization* of *immigrants* vis-à-vis the category of *Swedish*; the *stigmatization* of *immigrant* neighborhoods; and the negative *evaluation* of these neighborhoods through a biased formal and official assessment of value. We identify two additional processes: *resignification* in which ethnic *Swedish* people are reaffirmed as the symbolic center of Sweden who should be the focus of COVID planning; and *inversion* in which explanations of COVID inequality are inverted and, instead of something to be explained and addressed, *immigrants*’ risk is blamed on immigrants and used as an acceptable explanation for Sweden’s higher death rates.

These cultural processes are likely to be relevant for other cases. In our case, the processes compounded one another (e.g. racialization enables but does not cause inversion, stigmatization and the resulting segregation facilitate resignification) even if they did not appear in a strict sequential path. Because of the iterative character of cultural processes, we would imagine a range of combinations that would play out differently in different contexts with varying resonance and significance rather than a uniform sequence.

By questioning taken-for-granted explanations for Sweden’s lax approach to the pandemic (e.g. trust in government) and conventional explanations for inequality (e.g. socioeconomic inequality), we develop an explanatory framework rooted in cultural processes of inequality. But this problem is not confined to Sweden. Immigrants from different sending nations had elevated COVID-19 risk in many nations (Bentley, 2020; Greenaway et al., 2020; Yu, 2021). Examining cultural processes of inequality can shed crucial light on health inequalities in other contexts and related to other groups (Clair et al., 2016).

Recognition gaps open up new avenues for intervention. The recognition gaps we identify in this article are practical and solvable. Public health planning can anticipate and address health risks resulting from racialization, stigmatization, and resignification. Evaluation and inversion can be addressed through assessments of bias in policy and policy implementation. The iterative nature of the cultural processes of inequality means that intervening in one process can also have a positive impact via other processes. For example, addressing the resignification of the Swedish majority through campaigns fostering recognition of the equal social worth and social membership of immigrants can also facilitate de-stigmatization of immigrant spaces and decrease the risk of subsequent recognition gaps arising through biased standards for evaluation. Taking a cultural turn in the study of COVID inequality demonstrates the centrality of recognition gaps for persistent and emergent social inequality, and the promise of recognition efforts in the pursuit of more just and equal societies.
